# From 2D to 3D Bioprinted In Vitro Breast Cancer Model: A Comparative Study of Proliferation, Tissue Structure, and mTOR Signaling

**DOI:** 10.1002/mco2.70783

**Published:** 2026-05-26

**Authors:** Dorottya Moldvai, Gábor Petővári, Rebeka Gelencsér, Dániel Sztankovics, Risa Miyaura, Viktória Varga, Fatime Szalai, Kornélia Baghy, Ildikó Krencz, Titanilla Dankó, Anna Sebestyén

**Affiliations:** ^1^ Department of Pathology and Experimental Cancer Research Semmelweis University Budapest Hungary

**Keywords:** 3D bioprinting, breast cancer, mammalian target of rapamycin, model, preclinical

## Abstract

Three‐dimensional (3D) bioprinting offers a suitable in vitro preclinical model system to reduce or replace animal experiments; however, published studies are difficult to compare. In this study, we characterized growth dynamics, tissue architecture, and mammalian target of rapamycin (mTOR) pathway activity in a 3D bioprinted breast carcinoma model of T47D cell line and compared these features with conventional two‐dimensional (2D) monolayer cultures. Tissue‐mimetic structures (TMSs) were generated by 3D bioprinting and analyzed for cell viability, proliferation, autophagy, and apoptosis, as well as the expression of cell–cell and cell–extracellular matrix (ECM) adhesion proteins. In addition, mTOR pathway activity and responsiveness to mTOR inhibitors (rapamycin and ipatasertib) and chemotherapeutic agents (cisplatin) were assessed. The bioprinted TMSs remained viable for up to 3 weeks and developed a tissue‐like architecture characterized by heterogeneous marker expression (β‐catenin, E‐cadherin, N‐cadherin, fibronectin, and syndecan) and complex cellular organization. Compared with 2D monolayer cultures, 3D TMSs exhibited reduced mTOR signaling activity, which led to significantly decreased sensitivity to mTOR inhibition. These findings indicate that 3D bioprinted breast cancer models recapitulate key structural and signaling features of in situ tumors more accurately than 2D systems, highlighting their potential value for preclinical drug testing and mechanistic studies.

## Graphical abstract

A 3D bioprinted breast cancer model was created to more accurately reproduce in vivo tumor characteristics compared to conventional 2D cultures. Distinct cell adhesion patterns, reduced mTOR signaling, and altered drug responses were observed under 3D conditions. This model is proposed to provide more reliable data for preclinical cancer research.

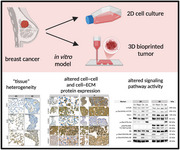

## 1 Introduction

Despite numerous successful animal experiments, many drug candidates fail in clinical phases. On average, only 16% progress from Phase 1 to approval, with oncology having the lowest success rate (8.3%) and ophthalmology the highest (29.5%) [1]. Safety concerns also cause post‐approval withdrawals; between 1990 and 2009, 4.2% of new drugs were withdrawn due to safety or risk–benefit issues [2, 3]. Preclinical studies play a critical role in selecting candidates [4], yet they often fail to predict human efficacy, wasting time, money, and animal lives. Many potentially effective molecules may also be discarded too early. One major reason for the high failure rate may be the reliance on two‐dimensional (2D) cell (mono)cultures in preclinical research, which fail to replicate cell–cell and cell–extracellular matrix (ECM) interactions, morphology, polarity, and complexity [5]. In vivo models include tissue complexity, but are limited by species differences, ethical concerns, and poor predictive value [6, 7, 8]. These shortcomings, combined with regulatory efforts to reduce animal use, have driven the development of alternative models [9]. Since April 2023, the European Union (EU) has prohibited animal testing for cosmetics and the sale of products with newly tested ingredients [10]. Although no equivalent regulations exist for pharmaceutical development, both the European Medicines Agency (EMA) and the U.S. Food and Drug Administration (FDA) emphasize reducing animal use (3R principles) and accelerating the development of new in vitro models [11, 12, 13]. While three‐dimensional (3D) bioprinting holds strong potential to replace animal testing, many protocols still rely on animal‐derived components, such as serum supplements (e.g., fetal bovine serum [FBS]) or hydrogel additives (e.g., gelatin, Matrigel). These raise concerns related to ethical issues and reproducibility (undefined batch‐to‐batch variability, microbiological contamination). However, promising alternatives are increasingly available, including serum‐free or chemically defined media, human platelet lysate, sericin protein, as well as plant‐based and synthetic hydrogels, which may further enhance the ethical and scientific rigor of future 3D bioprinted models [14].

There is a growing need for better preclinical disease models [15, 16]. Over the past 15 years, the paradigm has shifted toward 3D cell culture methods: organoids, spheroids, matrix gel‐embedded cultures, magnetic levitation, and 3D bioprinting [17, 18, 19]. Spheroids were the dominant 3D cell culture method in the past, but organoids and 3D bioprinting have gained popularity over the last decade. While only ∼8% of spheroid publications are reviews, this proportion is much higher for newer methods: ∼40% for 3D bioprinting and ∼30% for organoids (Figure S1). 3D bioprinting uses bioinks containing living cells and biomaterials to create multicellular, tissue‐like structures layer by layer. These models reproduce cancer hallmarks, including altered metabolism, vascularization, invasion, and metastasis [20, 21, 22]. Unlike spheroids and organoids, bioprinting allows greater reproducibility, spatial control, and standardization [23]. It supports long‐term growth, enables deliberate tissue design with multiple cell types, and is suitable for high‐throughput screening [24]. Importantly, it can incorporate patient‐derived cells, providing personalized cancer models that better mimic in situ tumors and drug responses than 2D cultures or xenografts [25]. However, only a few studies have directly compared signaling pathways and drug responses in 2D versus 3D culture conditions [26, 27, 28, 29].

Cell adhesion structures play a pivotal role in cell–cell communication, adhesion, and cellular stiffness: they regulate diverse cellular functions, including survival and migration through downstream signaling pathways [30]. For example, β‐catenin, E‐cadherin, and N‐cadherin contribute to cell adhesion and tissue stabilization, influence Wnt signaling as transcriptional regulators, and enhance tumor cell survival [31, 32]. Fibronectin and syndecans play key roles in establishing cell–ECM interactions, migration, and matrix organization, but their role in progression requires further clarification [33]. Changes in cell adhesion and ECM can also reshape cellular signaling through modulating the phosphatidylinositol‐3‐kinase (PI3K)/protein kinase B (Akt)/mammalian target of rapamycin (mTOR) pathway [34, 35], which regulates survival, growth, and proliferation [36, 37, 38]. Dysregulation of this pathway is the most common contributor to cancer development [39, 40]. c‐Jun N‐terminal kinase (JNK) and tuberous sclerosis complex (TSC) pathways regulate mTOR activity by modulating the stability, activity, and half‐life of its components. In 3D cell cultures, this regulatory network differs from 2D due to more complex interactions, influencing responses to stress and nutrient availability [41, 42]. Evidence shows that gene expression profile, mTOR activity, and drug sensitivity differ between 2D and 3D models [22, 43, 44], highlighting the need for further research. Proliferating, quiescent, and dying cells coexist within 3D spheroids [45] or other tissue‐like 3D structures, which may also contribute to the differences and complexity of the models.

In this study, 3D bioprinted tissue‐mimetic structures (TMSs) were fabricated using an mCherry‐labeled T47D cell line. Widely used proliferation assays have been validated on 3D bioprinted TMSs based on constitutive mCherry fluorescence. Differences in cell–cell/cell–ECM‐related protein expression, PI3K/Akt/mTOR pathway activity, and drug sensitivity between 2D cell cultures and 3D bioprinted TMSs were analyzed. Generally, 3D bioprinted TMSs displayed lower mTOR pathway activity, which may account for their reduced sensitivity, along with increased in situ heterogeneity and alterations in the expression and localization of cell–cell and cell–matrix adhesion proteins.

## 2 Results

### 2.1 Development and Validation of 3D Bioprinted TMSs for Cellular Dynamic Analysis

**FIGURE 1:**
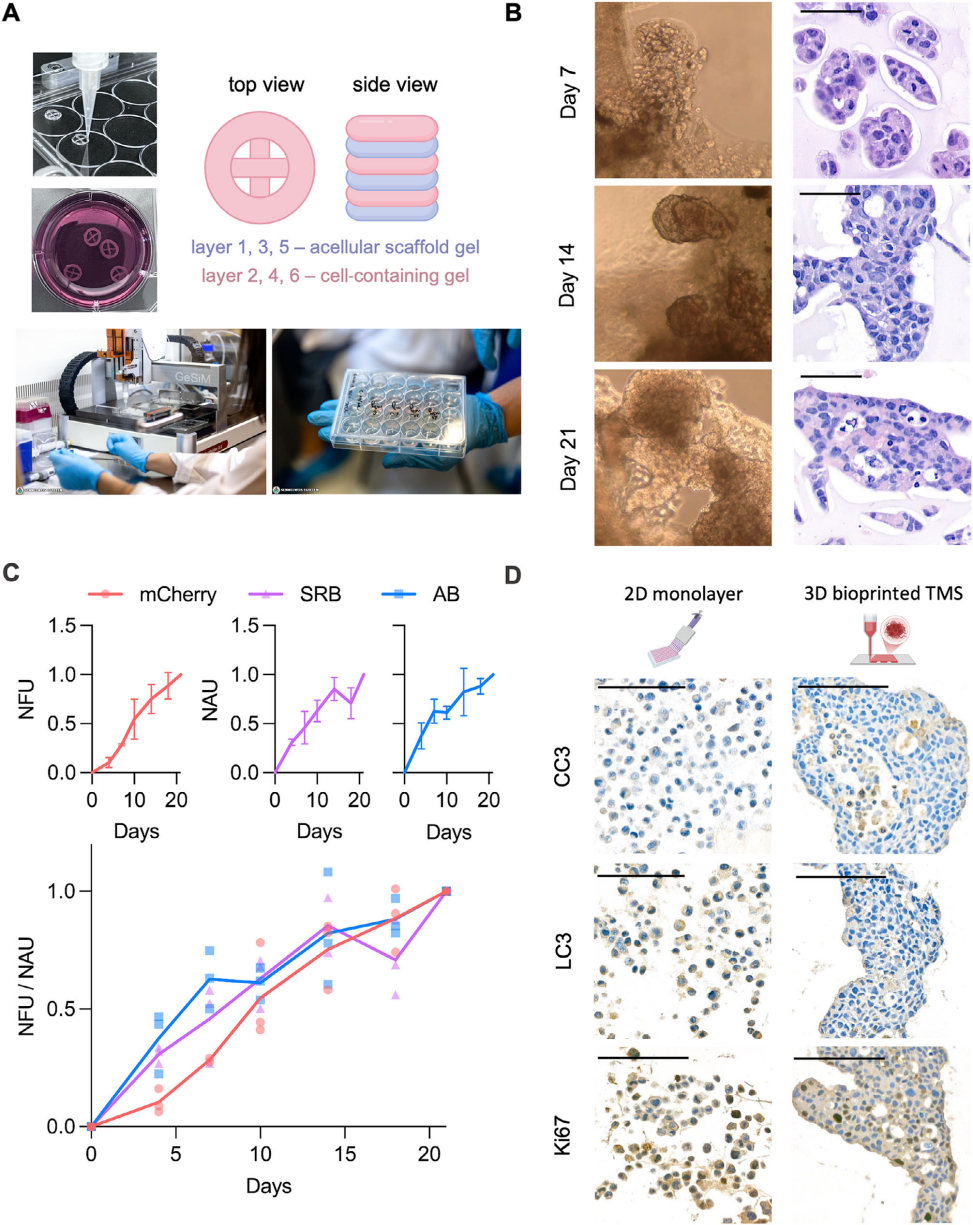
Validation of tissue formation and growth of 3D bioprinted T47D TMSs. (A) Schematic of the 3D bioprinted TMSs consisting of six alternating layers of cell‐containing gel and acellular scaffold gel, and the process of live‐cell bioprinting. (B) Tissue formation was monitored through light microscopy and hematoxylin–eosin (H&E) staining at days 7, 14, and 21 (4x magnification; scale bar: 50 µm). (C) Growth evaluation using constitutive mCherry fluorescence, AB, and SRB assays. No significant differences were observed (paired *t*‐test, *p > 0.05*, NFU ‐ normalized fluorescence unit, NAU ‐ normalized absorbance unit). (D) Apoptosis (CC3, cleaved caspase‐3), autophagy (LC3, light chain 3), and proliferation (Ki67) were analyzed via IHC in FFPE sections of cell blocks derived from 2D cell cultures and 3D bioprinted TMSs. (DAB chromogen; hematoxylin counterstain; scale bar: 100 µm).

The 3D bioprinted TMSs, fabricated using mCherry‐transfected T47D breast carcinoma cells, consisted of six alternating layers of cell‐containing gel and acellular scaffold gel (Figure 1A). The 3D bioprinted TMSs were maintained in vitro for 21 days, tissue formation was monitored using light microscopy, and formalin‐fixed paraffin‐embedded (FFPE) sections were stained with hematoxylin–eosin (H&E). Tissue formation was observed 1 week post‐printing, with extensive tissue‐like structures by day 21 (Figure 1B). Proliferation was evaluated using constitutive mCherry fluorescence and two assays, Alamar Blue (AB) for metabolic activity and sulforhodamine B (SRB) for total protein content. No significant differences were observed in the proliferation results between the assays when compared to the baseline mCherry fluorescence. Our results suggest that the AB and SRB assays are suitable for monitoring the growth of 3D bioprinted TMSs, which exhibited continuous growth over a 3‐week period (Figure 1C). Immunohistochemistry (IHC) analysis of 2D cell cultures and 3D bioprinted TMSs included stainings for cleaved caspase‐3 (CC3, apoptosis), light chain 3 (LC3, autophagy), and Ki67 (proliferation). In the 3D bioprinted TMSs, apoptosis was concentrated in the central regions, whereas it was infrequent in 2D cultures. The expression of autophagy‐related proteins was more pronounced in 2D cell cultures, indicating a potentially higher autophagic activity compared to 3D bioprinted TMSs. Ki67 staining in 2D cell cultures showed nearly uniform proliferation, whereas in 3D bioprinted TMSs, fewer proliferating cells were heterogeneously distributed throughout the “tissue”‐like structure (Figure 1D).

### 2.2 Expression of Cell Adhesion Proteins in 3D Bioprinted TMSs

**FIGURE 2:**
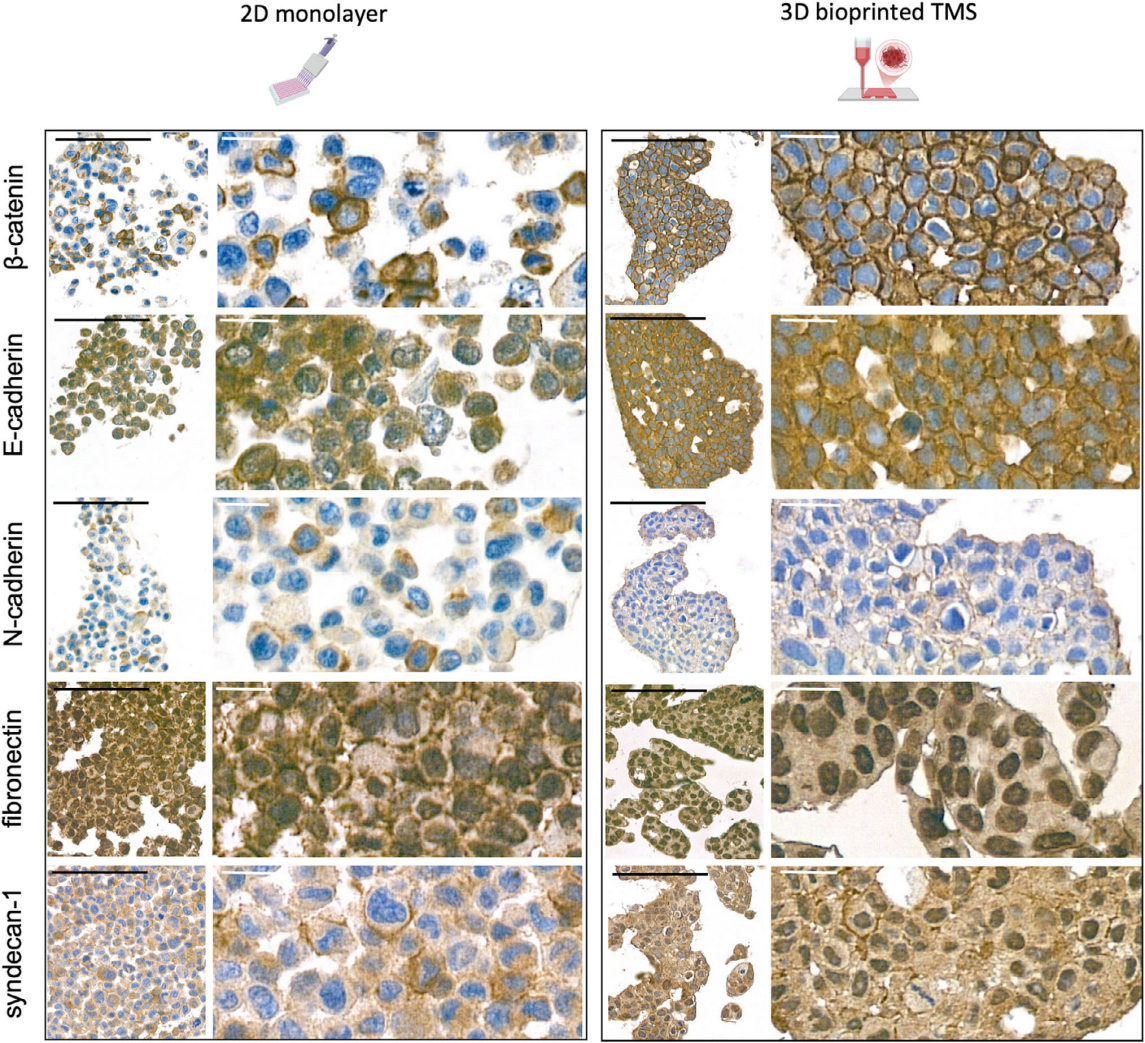
Protein expression regarding cell–cell/cell–ECM adhesion differs between 2D cell cultures and 3D bioprinted TMSs. IHC analysis of membrane and cell adhesion proteins was evaluated: β‐catenin, E‐cadherin, N‐cadherin, fibronectin, and syndecan‐1. (DAB chromogen; hematoxylin counterstain; scale bars: black = 100 µm, white = 20 µm).

IHC analysis of cell adhesion and ECM proteins revealed differences between the two model systems and highlighted a closer resemblance of the 3D bioprinted TMSs to clinical breast cancer (ductal carcinoma) tissue. In 3D TMSs, β‐catenin exhibited strong staining across cell membranes throughout the entire structure, whereas in 2D cell cultures, membrane positivity was limited to a few scattered cells. This distribution in 3D TMSs closely mirrors the localization patterns typically observed in situ, in contrast to the less representative organization seen in 2D cultures. E‐cadherin demonstrated robust and specific membrane localization in 3D TMSs, whereas in 2D cultures, strong staining was observed, but with a less defined pattern and lower expression, again deviating from the pattern commonly detected in tumor tissue. N‐cadherin expression further supported this distinction. While some breast cancer tissues are negative for N‐cadherin, cases with positive expression usually display membrane localization over extended tumor areas. This pattern was recapitulated in 3D TMSs, which exhibited pronounced and specific membrane staining across the entire structure. In contrast, in 2D cultures, N‐cadherin expression was weak and limited to a few scattered cells. Fibronectin displayed a more complex and heterogeneous localization pattern. In 3D TMSs, fibronectin exhibited a pronounced perinuclear/nuclear staining pattern, whereas in 2D cultures, it was predominantly membrane‐associated. Notably, both localization patterns could be observed in situ, where membrane‐dominant as well as internalized fibronectin staining has been reported in patient samples. Syndecan‐1 expression was also distinct between the two systems: in 3D TMSs, weak but homogeneous cytoplasmic and nuclear staining combined with a more pronounced membrane positivity was observed. In contrast, in 2D cultures, syndecan‐1 displayed a weaker cytoplasmic signal, but retained detectable membrane positivity. Importantly, in situ tumor samples similarly exhibit both membrane‐associated and internalized syndecan‐1 localization (Figure 2).

### 2.3 Decreased mTOR Pathway‐Related Protein Expression in 3D Bioprinted TMSs

**FIGURE 3:**
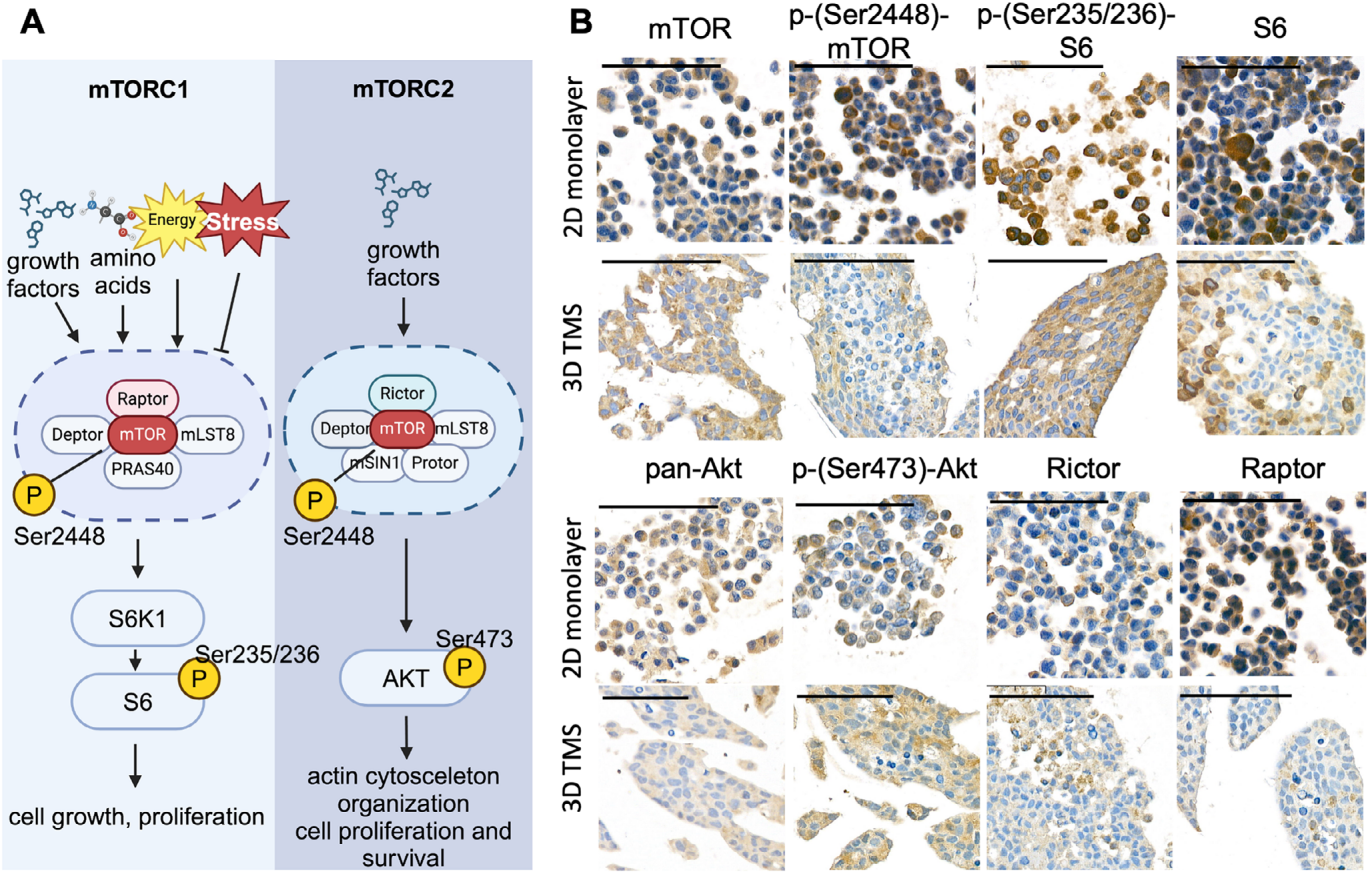
mTOR pathway‐related protein expression differs between 2D cell cultures and 3D bioprinted TMSs. (A) Simplified schematic of mTORC1 and mTORC2 signaling pathways created with BioRender. (B) Comparison of mTOR pathway markers (mTOR, p‐(Ser2448)‐mTOR, S6, p‐(Ser235/236)‐S6, pan‐Akt, p‐(Ser473)‐Akt, Rictor, Raptor) via IHC in 2D and 3D systems (DAB chromogen; hematoxylin counterstain; scale bar: 100 µm).

The mTOR signaling pathway is essential for the regulation of various cellular physiological processes, including growth, proliferation, and metabolism (Figure 3A). Generally, lower expression levels of mTOR‐related proteins were observed in 3D bioprinted TMSs than in 2D cell cultures. While total protein levels (mTOR, S6, pan‐Akt) showed no visually significant differences, phosphorylated proteins, particularly p‐(Ser2448)‐mTOR and p‐(Ser235/236)‐S6, exhibited reduced staining intensity and fewer positively stained cells in 3D bioprinted TMSs. Interestingly, stronger phospho–protein staining was observed at the periphery of the 3D structures, suggesting more pronounced growth activity in these regions. In addition, p‐(Ser‐235/236)‐S6 showed a notably heterogeneous staining pattern, especially in 3D bioprinted TMSs (Figure 3B).

### 2.4 Quantitative Evaluation of Decreased mTOR Activity in 3D Bioprinted TMSs

**FIGURE 4:**
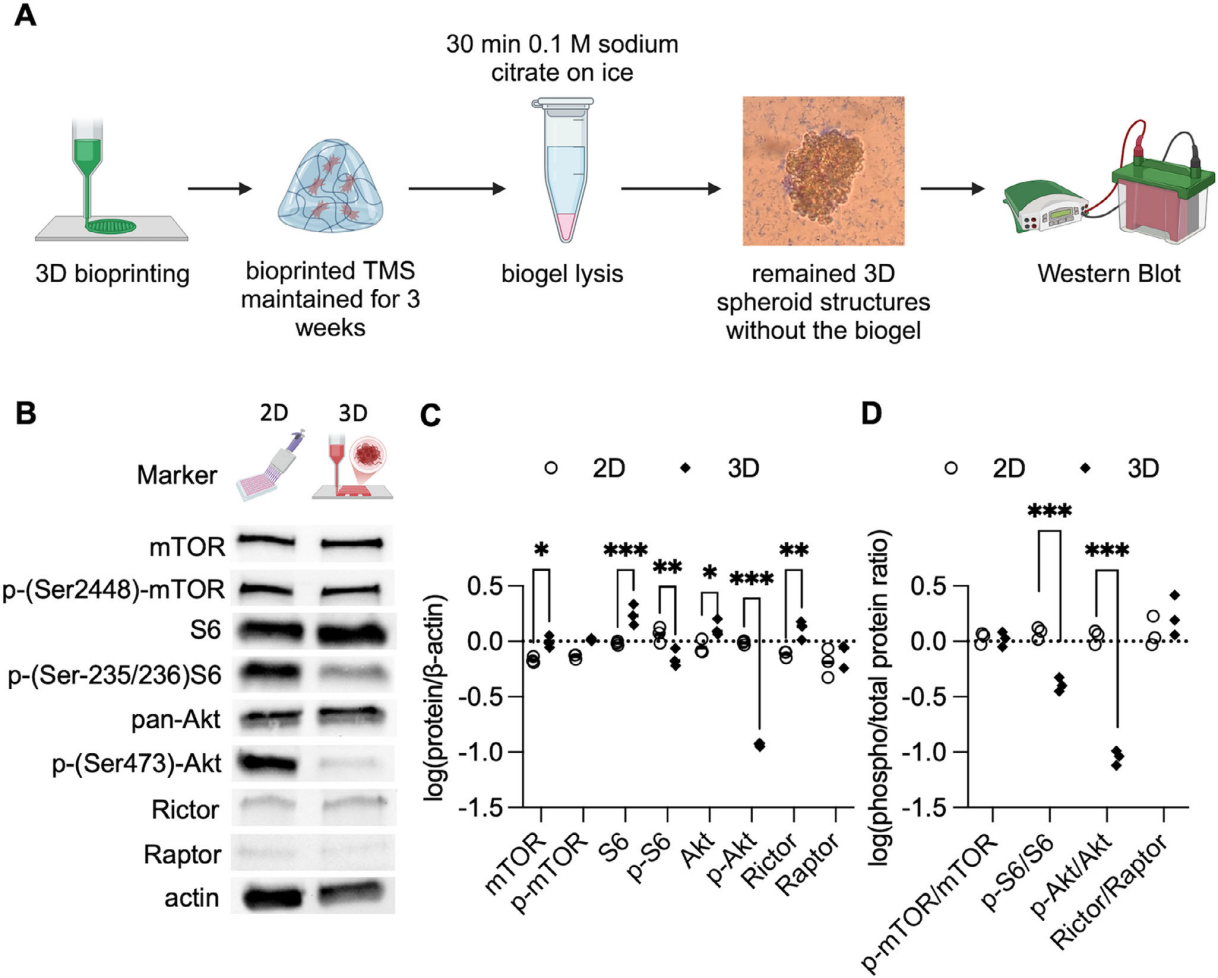
Quantitative comparison of mTOR pathway‐related protein expression between 2D cell cultures and 3D bioprinted TMSs through Western blot (WB) analysis. (A) Workflow showing the preparation of 3D bioprinted TMSs for protein analysis. (B) WB analysis of mTOR activity markers (mTOR, p‐(Ser2448)‐mTOR, S6, p‐(Ser235/236)‐S6, pan‐Akt, p‐(Ser473)‐Akt, Rictor, Raptor) in 2D cell cultures and 3D bioprinted TMSs. All measurements were performed on at least three parallel samples. (C) Densitometric quantification of protein expression levels, normalized to β‐actin, with statistical significance indicated (^*^ *p* < 0.05; ^**^ *p* < 0.01; ^***^ *p* < 0.001; two‐way ANOVA with Fisher's LSD test). (D) Phospho‐to‐total protein ratio analyses for key markers, highlighting differences in mTOR activity (^*^ *p* < 0.05; ^**^ *p* < 0.01; ^***^ *p* < 0.001; two‐way ANOVA with Fisher's LSD test).

To prepare 3D bioprinted TMSs for WB analysis, the surrounding hydrogel posed a challenge for electrophoresis. Sodium citrate digestion was performed on ice to dissolve the hydrogel, ensuring that cell–cell interactions and the structural integrity of the “tissue” were preserved. (Figure 4A). To compare mTOR activity between 2D cell cultures and 3D bioprinted TMSs, the expression of mTORC1 (p‐(Ser235/236)‐S6, S6, p‐(Ser2448)‐mTOR, mTOR, Raptor) and mTORC2 markers (p‐(Ser473)‐Akt, pan‐Akt, p‐(Ser2448)‐mTOR, mTOR, Rictor) were analyzed. Significantly higher expression levels of mTOR (0.99 vs. 0.68), S6 (1.75 vs. 0.96), pan‐Akt (1.32 vs. 0.89), and Rictor (1.30 vs. 0.77) were observed in 3D bioprinted TMSs compared to 2D cell cultures. In contrast, lower expression levels of p‐(Ser‐235/236)‐S6 (0.71 vs. 1.16) and p‐(Ser473)‐Akt (0.11 vs 0.97) were noted in 3D bioprinted TMSs (Figure 4B,C). The phospho‐to‐total protein ratios indicated decreased p‐S6/S6 (0.41 vs. 1.20) and p‐Akt/Akt (0.09 vs. 1.11) levels in 3D bioprinted TMSs, suggesting reduced mTOR activity. The Rictor‐to‐Raptor ratio showed no significant difference, implying that the overall quantity of mTOR complexes likely remained unchanged, although their activity was reduced under 3D conditions (Figure 4D). The differences observed in β‐actin levels across the model systems may be due to variations in cell morphology. In 2D cell cultures, cells exhibit a more spread shape, whereas in 3D conditions, they adopt a more compact, rounded morphology, which may be reflected in cytoskeletal differences.

### 2.5 Decreased mTOR Inhibitor Sensitivity in 3D Bioprinted TMSs

**FIGURE 5:**
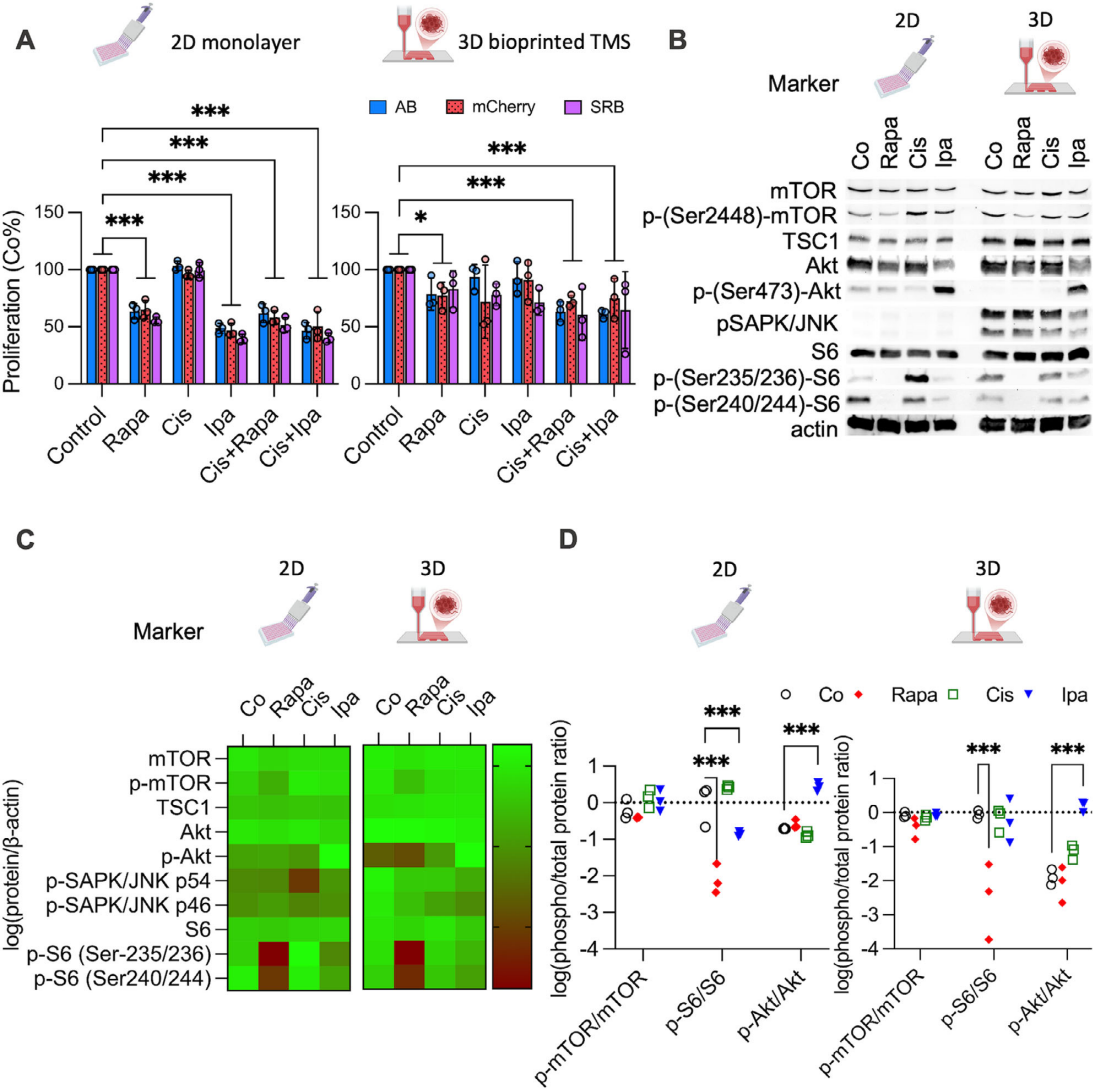
Reduced sensitivity to mTOR inhibitors was observed in 3D bioprinted tissue‐mimetic structures (TMSs) compared to 2D cell cultures. (A) Rapamycin (Rapa), cisplatin (Cis), ipatasertib (Ipa), and their combinations were tested using Alamar Blue (AB), sulforhodamine B (SRB) assays, and mCherry fluorescence, with results presented as percentage of control (^*^ *p* < 0.05; ^**^ *p* < 0.01; ^**^ *p* < 0.001; paired *t*‐test). (B) mTOR activity markers were analyzed by Western blot in 2D cell cultures and 3D bioprinted TMSs. (C) Densitometry heat maps show higher (green) and lower (red) protein expression. (D) Phospho/total protein ratios related to mTOR signaling were analyzed (^*^ *p* < 0.05; ^**^ *p* < 0.01; ^***^ *p* < 0.001; two‐way ANOVA with Fisher's LSD test).

The sensitivity of 2D cell cultures and 3D bioprinted TMSs to mTOR inhibitors was assessed. In vitro treatments included rapamycin (Rapa), cisplatin (Cis), ipatasertib (Ipa), and their combinations (Cis+Rapa; Cis+Ipa). Significant inhibition of proliferation was observed in 2D cell cultures following Rapa, Ipa, and their combination treatments, whereas resistance to Cis was observed. In contrast, 3D bioprinted TMSs demonstrated reduced sensitivity to Rapa and complete resistance to Ipa. In addition, in both in vitro models, the combination treatments were highly effective and showed Cis sensitization in 3D bioprinted TMSs (Figure 5A). Densitometric analysis revealed a significant decrease in p‐(Ser‐235/236)‐S6 expression in 2D cell cultures following Rapa (0.005 vs. 0.86) and Ipa (0.10 vs. 0.86) treatments. However, in 3D bioprinted TMSs, only Rapa reduced p‐(Ser‐235/236)‐S6 expression (0.003 vs. 0.78). Notably, Ipa treatment significantly increased p‐(Ser473)‐Akt expression in both 2D cell cultures (1.82 vs. 0.19) and 3D bioprinted TMSs (1.90 vs. 0.02). Higher baseline expression levels of TSC1 and pSAPK/JNK were observed in 3D bioprinted TMSs compared to the 2D cell cultures (TSC1: 0.78 vs. 0.45; pSAPK/JNK p54: 1.35 vs 0.12; pSAPK/JNK p46: 1.06 vs. 0.13). (Figure 5B,C). Densitometric analysis of phospho‐to‐total protein ratios revealed that Rapa and Ipa significantly reduced the p‐(Ser‐235/236)‐S6/S6 ratio in 2D cultures (Rapa: 0.01 vs 1.46; Ipa: 0.13 vs. 1.46) compared to controls. However, in 3D bioprinted TMSs, only Rapa reduced the p‐(Ser‐235/236)‐S6/S6 ratio (0.01 vs. 0.90), while Ipa had no significant effect. Furthermore, Ipa markedly increased the p‐(Ser473)‐Akt/Akt ratio under both conditions (2D: 2.75 vs. 0.20; 3D: 1.60 vs. 0.01) (Figure 5D), consistent with the ATP‐competitive Akt inhibitory effects of Ipa. All detected differences in sensitivity and expression highlight that combination treatments show more stable and pronounced effects in both 2D and 3D bioprinted in vitro models. Despite significant differences in the efficacy of monotherapies and associated signaling pathway alterations, combination treatments demonstrated consistently stable and effective therapeutic effects in both 2D and 3D in vitro models.

## 3 Discussion

In this study, an in vitro 3D bioprinted breast cancer model was established and characterized using mCherry‐transfected T47D cell line, enabling real‐time monitoring of growth and validation of standard proliferation assays. Histopathological analyses revealed tissue‐like architecture with central apoptosis, heterogeneous proliferation, and distinct adhesion protein expression patterns resembling in situ breast carcinoma. Compared with 2D cultures, the 3D bioprinted TMSs exhibited reduced mTOR pathway activity, altered regulation of autophagy‐ and stress‐related markers, and significant resistance to mTOR inhibitors, while combination treatments maintained efficacy.

Tissue engineering and live‐cell 3D bioprinting are promising methods for creating functional 3D models for various applications, including cancer drug development [46], the fabrication of biomimetic prostheses [47], and even the construction of transplantable organs [48]. Despite the growing interest in bioprinting, the field remains dominated by review‐type publications (approximately 40%), suggesting that the scientific community is more focused on summarizing existing knowledge than performing new experimental studies [49]. Several factors may have contributed to this phenomenon. During the COVID‐19 pandemic, the focus shifted from experimental research to the publication of review articles, leading to a decreased influx of research papers. In addition, the pressure to publish is driven by requirements related to peer‐review processes, national funding systems, and academic criteria for obtaining university degrees and PhD qualifications, impacting various research groups. Although review articles are crucial, there is a need for a more robust influx of original research to drive the field forward.

The aim of 3D bioprinting is to model cell–cell interactions, the tumor microenvironment (TME), and tissue heterogeneity, and to mimic in situ tumor behavior and drug sensitivity more accurately. Several variables, such as hydrogel properties (e.g., composition and viscosity), printing conditions (pressure, temperature, needle size, speed, design, cross‐linking, etc.), and maintenance conditions (medium type, maintenance duration), make standardization and high‐throughput applications difficult. The presented data collection of 3D bioprinted breast cancer models represents wide diversity across studies, where different bioinks, cell lines, and shapes are used. Moreover, there are no standardized post‐printing and treatment timelines for 3D bioprinted cancer model experiments [24]. Therefore, inter‐study comparisons remain challenging, underscoring the need for standardization to unlock the full potential of 3D bioprinting as a reproducible alternative to xenografts or other in vitro systems.

In our recent study, we developed a 3D bioprinted breast cancer model using a stable mCherry‐transfected cell line (T47D). The 3D TMSs were maintained for 21 days, during which their growth was monitored through the constitutive fluorescence of mCherry. Additional proliferation assays (AB and SRB) were validated using mCherry fluorescence to confirm their applicability to 3D bioprinted TMSs. Our results highlight that mCherry‐transfected cells will enable the development of more complex models in the future, facilitating co‐culture experiments with other cell types and allowing distinct monitoring of cancer and cancer‐associated cell growth.

Based on histopathological examination, apoptosis was observed in the central regions of the 3D TMSs, while Ki67 staining was heterogeneous, indicating a relatively low number of cycling tumor cells. This pattern closely resembles the in situ behavior of breast cancer [50]. Interestingly, the autophagy‐related protein LC3 showed higher expression in 2D cell culture, which suggests that cells cultured in 2D conditions may experience stress that activates autophagy, despite the homogeneous and virtually unlimited supply of nutrients and oxygen. A study by Follo et al. reported that decreased or increased autophagy is cell type rather than 2D/3D environment‐dependent [51].

In 2D cultured cells, β‐catenin, E‐cadherin, and N‐cadherin were diffusely distributed in the cytoplasm, in correlation with weak or absent cell–cell adhesion [30, 52]. These proteins are essential for tissue stabilization, and through nuclear translocation, β‐catenin signaling enhances tumor cell survival in the tissue environment [31, 32]. The observed alterations in 3D TMSs, including adhesion‐mediated increase in β‐catenin and robust membranous E‐ and N‐cadherin expression [53], might contribute to signaling alterations, aggressiveness, and metastatic potential [54]. The consequential increase in mTOR inhibitor resistance in our 3D structures was consistent with previously published findings [55]. Changes in the expression patterns of ECM proteins, including fibronectin and cell surface heparan sulfate proteoglycan (HSPG) syndecans, may also correlate with oncogenic transformation [33]. Loss of cell‐surface fibronectin has been associated with oncogenic transformation, as previously suggested. Moreover, recent findings indicate that fibronectin migrates to the nucleus during epithelial‐to‐mesenchymal transition (EMT) progression [56], potentially contributing to Rapa resistance [57]. In addition, the cytoplasmic rather than membrane localization of syndecan‐1 in 3D bioprinted TMSs could have a possible transcriptional regulatory function [58, 59], which may influence tumor progression and treatment resistance, similar to its association with poorer prognosis in human breast cancer [60, 61]. According to one study, the less well differentiated a tumor is and the higher its grade, the more strongly it internalizes syndecan‐1, which is consistent with our observed findings, as the drug sensitivity of the 3D cell culture decreases [62].

In parallel with the adhesion and ECM changes, we also observed changes in the elements of crossroads in the signaling network. The mTOR‐related phospho‐to‐total protein ratios were significantly lower in 3D bioprinted TMSs, even without treatment, indicating reduced mTOR activity in these models. In addition, pSAPK/JNK Thr183/185 and TSC1 showed higher expression in our 3D bioprinted TMSs than in 2D cell cultures, which can be attributed to several factors in a complex 3D environment. JNK has Janus‐faced activity that regulates both cell death and survival and is activated by various stress signals [41]. This pathway interacts with mTOR and influences its regulatory functions. JNK activation can lead to mTOR inhibition [63], thereby influencing drug sensitivity, as observed in our experiments. Other published results based on a cholangiocarcinoma model also highlighted that the 3D bioprinted environment may lead to upregulated TSC1 as a feedback mechanism to modulate mTOR signaling [64]. The activation of stress response pathways may lead to different regulation of the JNK and mTOR pathways compared with simpler 2D cultures. The built 3D architecture in 3D bioprinted TMSs, together with the development of nutrient and oxygen gradients, inner hypoxic zones [65], enhanced cell–cell interactions, and matrix stiffness, shapes the complex TME, tissue formation, and drug resistance. These multiple interacting factors are highly important in modeling in situ tumors, since numerous influences act on them simultaneously, defining their properties in a complex interplay.

The additional stress in the formation of “tissues” and the adaptation mechanisms (the above‐discussed complex alterations) lowered the therapeutic sensitivity in in vitro treatments of the 3D bioprinted TMSs. In addition to the previously observed Cis resistance in 2D cultures, Rapa and Ipa resistance were detected in 3D cultures. In our study, Ipa treatment significantly increased the levels of p‐(Ser473)‐Akt in both 2D cell cultures and 3D TMSs, which can be explained by the mechanism of Ipa and other ATP‐competitive Akt inhibitors [66, 67, 68]. Ipa binds to the active site of Akt, preventing phosphatases from accessing these sites, which leads to an increase in Akt phosphorylation. However, despite the increase in Akt phosphorylation, the downstream pathway remains suppressed during Ipa treatment [69]. In addition, in both 2D and 3D bioprinted in vitro models, the combination treatments were highly effective and showed Cis sensitization.

The detected expression changes of cell–cell and cell–ECM adhesion proteins with signaling network activity differences may explain the increasing tumorigenic potential and reduced drug sensitivity observed in 3D bioprinted structures. These new complex findings, along with our new 3D bioprinted in vitro breast cancer model, revealed altered expression, localization, and complex networking of several proteins generally associated with worse prognosis. These could influence the therapeutic sensitivity of complex 3D structures and can also be detected in human cancer tissues.

Our findings are consistent with those of a previous study by Riedl et al., who observed reduced mTOR signaling in 3D‐cultured spheroids compared to 2D cultures of colon cancer cell lines. In contrast to our results for breast carcinoma, their research demonstrated a greater response to drugs targeting these pathways in their 3D spheroid models [43]. The observed differences could be tumor and 3D model‐type dependent. A previous study also suggested that the drug responses and protein expression profiles of spheroid cultures resemble those of 2D models more closely, whereas other tissue‐like in vitro cultures (e.g., 3D bioprinting) are better suited for modeling in situ and/or in vivo tumor contexts [22]. In addition, other published data support our findings in different carcinoma cell lines. Weigelt et al. observed a reduction in Akt phosphorylation in 3D laminin‐containing gel‐embedded cultures of other HER‐2‐positive breast cancer cell lines in targeted drug experiments. Their study also demonstrated altered activation of the PI3K‐Akt and Ras‐MAPK pathways in 2D and 3D environments [70]. Similarly, Frtús et al. reported decreased pmTOR activity in 3D collagen scaffold cultures of HEPG2 and Alexander cell lines compared to that in 2D monolayer cultures (hepatocellular carcinoma) [71]. Our recent pioneering attempts aimed to develop long‐term 3D bioprinted breast cancer cultures with advanced characterization, highlighting the complex alterations in adhesion, ECM protein expression, signaling networks, and drug sensitivity within in vitro cultured tissues. We have successfully created and characterized this novel in vitro breast carcinoma model to facilitate standardization (e.g., defined conditions and assay validation) and to comprehensively compare the differences between the new 3D bioprinted model and traditional 2D cultures.

Based on our findings, 3D bioprinted TMSs offer significant advantages over traditional 2D cell cultures in preclinical drug development because of their ability to replicate the structural and functional complexity of the TME. Unlike 2D models, which lack the spatial organization and ECM interactions, 3D bioprinted tumors enable the study of cell–cell and cell–ECM dynamics, as well as signaling network heterogeneity and therapeutic response that are critical to tumor biology and drug development. However, this study has limitations that should be considered when interpreting the results. First, the study did not include clinical samples or in vivo experiments. While this approach allows controlled assessment of structural and signaling differences between 2D and 3D culture systems, it restricts the direct translational relevance of the findings. Second, the bioink used in this study contained a relatively high concentration of alginate, which limits cellular motility and migration. As a result, certain cell–cell and cell–ECM interactions may be constrained under these conditions. It is likely that alternative hydrogel compositions would differentially influence cellular organization, matrix interactions, and signaling behavior, and this represents an important area for future investigation. Finally, the present study focused on a single (but special, endogenously mCherry‐expressing) breast cancer cell line evaluated across multiple model systems. Consequently, the conclusions are more relevant to model development and methodological comparison than to broader tumorbiological or molecular generalization. Further refinement of 3D bioprinted cancer models could include the integration of additional cell populations to more closely mimic the TME and its complex cellular interactions (tumor–stroma crosstalk, immune evasion, and therapeutic resistance) [72]. In parallel, the development of standardized bioinks, printing protocols, and assay platforms will be essential for enabling reproducibility and scalability. Establishing these standardized approaches could position 3D bioprinting as a robust platform for high‐throughput drug screening and preclinical testing, thereby increasing its translational impact.

## 4 Conclusion

Our new 3D bioprinted breast cancer model demonstrates significant potential for advancing preclinical drug development by more accurately mimicking the TME and intracellular signaling alterations than traditional 2D cell cultures. Despite challenges, such as the lack of standardization in bioinks and printing conditions, variations in experimental timelines, and difficulties in inter‐study comparisons, the developed 3D bioprinted model exhibits enhanced translational relevance. This provides insights into the mechanisms underlying the differences in stress responses and drug sensitivity compared to 2D cultures. As initially validated, traditional proliferation assays remain critical for studying tumor behavior and drug responses, and they can be applied to 3D bioprinted cultures. Our study is the first to describe alterations in key signaling proteins, cell growth dynamics, ECM components, and cell‐adhesion molecules when comparing 2D and 3D bioprinted culture conditions of breast cancer. These findings underscore the fact that the 3D bioprinted environment induces complex cellular changes, which may contribute to the observed drug response discrepancies. Our results highlight the necessity of transitioning from 2D to 3D bioprinted cancer models for future basic research and preclinical drug development. Further refinement and development of this 3D bioprinted breast cancer model, such as increasing its complexity by incorporating additional cancer‐associated cells, may revolutionize cancer drug development by improving success rates and reducing costs.

## 5 Materials and Methods

### 5.1 Literature Review

Literature on tumor models used in cancer biology research was examined in the PubMed database using the search terms: “cancer model” AND “3D” AND (“organoid” or “bioprinting” or “spheroid”). The query was made on February 5, 2025, and focused on data from 2010 to 2024, and only articles with at least an English‐language abstract were included. The results were visualized in a cumulative bar chart, illustrating both the percentage distribution of the results and the growth in the number of publications over time. The ratio of review articles to original research papers was also analyzed for spheroids, organoids, and bioprinted models across the three types of 3D tumor models.

### 5.2 Cell Culture and In Vitro Reagents

For in vitro experiments, mCherry‐transfected luminal A‐type (ER+, PR±, HER2−, Ki67 low) T47D breast cancer cells (a kind gift from Csilla Özvegy‐Laczka, Institute of Enzymology, Hungarian Academy of Sciences, Budapest; obtained from the American Type Culture Collection [ATCC] and transfected with the pRRL‐EF1‐mCherry‐WPRE [Addgene] lentiviral vector [73]) were cultured and treated in RPMI‐1640 medium (Biosera, France) at 37°C and 5% CO_2_. The medium was supplemented with 10% FBS (Biosera), 2 mM L‐glutamine (Biosera), and 100 U mL^−1^ penicillin–streptomycin (Biosera). For in vitro treatments, Rapa (50 ng mL^−1^; Merck), Ipa (0.5 µM; Selleckchem), Cis (1 µM; Accord), and their combinations (Cis+Rapa; Cis+Ipa with the same concentrations as monotherapy) were used. Rapa is an allosteric inhibitor of mTORC1, Cis is a cytotoxic agent, and Ipa is an ATP‐competitive pan‐Akt inhibitor. The cell line passage number was between P+10 and P+25 in all experiments. An appropriate dilution of vehicle was used as the control group. *Mycoplasma* contamination was routinely checked using multiplex PCR during our working period.

### 5.3 Preparing Hydrogels and Cross‐Linking Agent for 3D Bioprinting

For 3D bioprinting, two types of bioinks were prepared: a less viscous cell‐containing gel consisting of 3% alginate (Merck‐Sigma‐Aldrich, Germany) and 1% gelatin (Merck‐Sigma‐Aldrich), and a stiffer acellular scaffold gel composed of 11% methylcellulose (Merck‐Sigma‐Aldrich) and 6% alginate [25]. To prepare these gels, alginate was dissolved in water and sterilized (120 kPa for 20 min). When the alginate solution remained warm, an appropriate amount of sterile gelatin or methylcellulose was carefully added. The acellular scaffold gel was loaded into the printer cartridges. After preparation, the acellular scaffold gel was allowed to rest in a refrigerator for at least 24 h before use. One hour before printing, it was warmed to room temperature. The cell‐containing gel was warmed to 37°C 1 h before use, and the T47D cells were gently mixed into it immediately prior to printing at a concentration of 10 million cells/mL and loaded into the cartridges. The two gels were applied to two different print heads.

### 5.4 3D Bioprinting of 3D TMSs

The 3D bioprinted TMSs were designed using GeSiM Robotics software and fabricated using an extrusion‐based bioprinter (Bioscaffolder 3.2, GeSiM, Germany) with two independently operated dispenser units. A grid‐like architecture was chosen for the bioprinted constructs. Since the diffusion distance of oxygen and nutrients in tissues is ∼100 µm, this design minimizes the risk of hypoxic or nutrient‐deprived zones in the absence of vasculature [74]. For proliferation assays, 5‐mm diameter structures were fabricated, consisting of six layers alternating between the cell‐containing and acellular scaffold gel layers, with a total height of 0.5 mm. For IHC staining, 10‐mm diameter structures consisting of 10 alternating layers with a total thickness of 1 mm were printed. Bioprinting parameters included: interlayer angle: 90°; infill distance: 1.5 µm; layer connection: outline plus; printing speed: 10 mm s^−1^; needle diameter: 110 µm (acellular scaffold gel) or 50 µm (cell‐containing gel); and pressure: 400 kPa (acellular scaffold gel) or 20 kPa (cell‐containing gel). After printing, the structures were stabilized using CaCl_2_ (Merck‐Sigma‐Aldrich; 200 mM; 2 min) and maintained in vitro in cell culture medium (RPMI‐1640). Medium changes were performed every 2–3 days to ensure proper nourishment and cell growth within the structures. The TMSs were maintained on p‐hema‐coated (poly(2‐hydroxyethyl methacrylate); Merck‐Sigma‐Aldrich) 6‐well plates (Sarstedt) to avoid cell adhesion.

### 5.5 Proliferation Assays

For 2D cell culture, T47D cells were seeded at a density of 3500 cells well^−1^ in a 96‐well plate (Sarstedt). After a 24 h incubation period, treatments were administered following medium exchange. For the 3D bioprinted structures, treatments were applied after a 1‐week pre‐culture period in a 6‐well plate, followed by medium exchange prior to treatment. Following a 72 h treatment period, cell viability and proliferation were assessed using AB (Thermo Fisher Scientific) and SRB assays. Proliferation assays for the TMSs were conducted in a 96‐well plate, with the TMSs being transferred directly into the wells immediately before the addition of AB. AB was diluted at a 1:10 ratio in the culture media. The long‐term proliferation of TMSs was measured from the moment of printing, and in this case, there was no pre‐culture period. Fluorescence measurements were taken after a 2‐h incubation using a Fluoroskan Ascent FL fluorimeter (570–590 nm; Labsystems International, Finland), and the data were analyzed using Ascent software (Labsystems International). For the SRB assay, treated cells were fixed with cold 10% trichloroacetic acid (Merck‐Sigma‐Aldrich; 60 min; 4°C), then thoroughly washed with distilled water, and subsequently dried. After drying, the cells were incubated with 0.4% SRB (Merck‐Sigma‐Aldrich; 15 min; RT, dissolved in 1% acetic acid). After washing with 1% acetic acid and subsequent drying, the protein‐bound dye was dissolved in 10 mM Tris buffer (Merck‐Sigma‐Aldrich), and the absorbance was measured at 570 nm using a LabSystems Multiskan RC/MS/EX Microplate Reader (Labsystems International). Each measurement was performed in sextuplicate and repeated thrice. Relative cell proliferation was calculated as a percentage of untreated control cells. For the treatments, a vehicle control was used, whereas cell‐free printed empty’ TMS structures were used as controls for the 3D bioprinting experiments.

### 5.6 Hematoxylin–Eosin Staining and Immunohistochemistry

H&E staining and IHC were performed on 3 µm‐thick sections prepared from FFPE blocks of both 3D bioprinted TMSs and 2D cultured cells. The 3D bioprinted TMSs were fixed in 10% formalin for FFPE block preparation and then stabilized in a 1% agar solution (4°C, 10 min). Agar disks were placed in cassettes, dehydrated, and embedded in paraffin. The 2D cell cultures were harvested using Triple X (Gibco, USA), suspended in a liquid agar solution, and fixed in formalin after solidification. After dehydration, the cultures were embedded in paraffin. After deparaffinization and blocking of endogenous peroxidase activity (periodic acid, sodium borohydride, and methanol‐H_2_O_2_), antigen retrieval was conducted using a pressure cooker (10 min, 10 mM citrate buffer, pH 6.0, or BOND‐PRIME Epitope Retrieval Solution 2, pH 9.0, Leica Biosystems, Germany). The slides were then incubated with primary antibodies (Table S1). Primary antibody detection was performed using a Novolink Polymer (Leica Biosystems) detection system. 3,3′‐Diaminobenzidine (DAB; Dako, Denmark) served as the chromogen, followed by hematoxylin counterstaining. Immunostained sections were scanned using SlideViewer 2.7 software (3DHistech, Hungary). IHC performed on 3D bioprinted TMSs showed no nonspecific background staining: an internal negative control was consistently present within the sections in regions containing only the scaffold material without cells, and all stainings were evaluated by a board‐certified pathologist to exclude nonspecific signals.

### 5.7 Western Blot

Because the bioink (alginate) interfered with measurement in the protein analysis of 3D bioprinted TMSs, it was removed prior to analysis. The structures were rinsed in phosphate‐buffered saline (PBS), incubated in sodium citrate solution (0.1 mM; 30 min; ice), followed by centrifugation (1000 rpm; 10 min; 4°C). This process dissolved the alginate gel while maintaining spheroid‐like cell aggregates, instead of a single‐cell suspension. These 3D cell structures were used for subsequent analyses. Cells or residual spheroids were rinsed with PBS and lysed in buffer containing 50 mM Tris, 10% glycerol, 150 mM NaCl, 1% Nonidet‐P40, 10 mM NaF, 1 mM PMSF, and 0.5 mM Na3VO4 at pH 7.5. Protein concentration was determined using the Bradford assay (Bio‐Rad, USA). Proteins were separated by SDS‐PAGE, transferred to polyvinylidene difluoride (PVDF) membranes, and probed with primary antibodies (Table S1), followed by incubation with biotinylated secondary antibodies and avidin‐HRP complex (Vectastain Elite ABC HRP Kit; Vector Laboratories, USA). Detection was performed with ECL reagent (Pierce ECL Western Blotting Substrate; Thermo Fisher Scientific, USA) and iBright Invitrogen FL1000 (Thermo Fisher Scientific). Densitometric analysis was conducted using ImageJ software to normalize the protein expression to β‐actin. Phosphorylated‐to‐total protein ratios (p‐mTOR/mTOR, p‐S6/S6, and p‐Akt/pan‐Akt) were calculated to assess mTOR kinase, mTORC1, and mTORC2 activity, respectively. The Raptor‐to‐Rictor ratio was used to determine the relative abundance of the mTORC1 and mTORC2 complexes. Each Western blot (WB) analysis was performed at least in triplicate. Expression values shown in brackets in the text related to WB results refer to mean values.

### 5.8 Statistical Analysis

For parametric data, a two‐sample *t*‐test was used to compare two groups, and two‐way ANOVA with Fischer's LSD test was applied for comparisons involving more than two groups. The D'Agostino–Pearson test was used to check for normality. Statistical analysis was conducted using GraphPad Prism version 10.4.1 (532) (GraphPad Software, USA). All experiments were performed using the data obtained from at least three independent measurements.

## Author Contributions

Conceptualization: Anna Sebestyén, Dorottya Moldvai, and Gábor Petővári. Data curation, formal analysis, project administration, and software: Dorottya Moldvai. Funding acquisition and resources: Anna Sebestyén. Investigation: Dorottya Moldvai, Risa Miyaura, Fatime Szalai, and Rebeka Gelencsér. Methodology: Dorottya Moldvai, Gábor Petővári, and Titanilla Dankó. Supervision: Anna Sebestyén, Gábor Petővári, Ildikó Krencz, Titanilla Dankó, and Kornélia Baghy. Validation: Dániel Sztankovics, Viktória Varga, and Ildikó Krencz. Visualization: Dorottya Moldvai. Roles/Writing: Dorottya Moldvai, Anna Sebestyén, Dániel Sztankovics, and Titanilla Dankó. All authors have read and approved the final manuscript.

## Funding

Recent research work at the Department of Pathology and Experimental Cancer Research, Semmelweis University, was funded by the National Bionics Program (Project No. ED_17‐1‐2017‐0009) from the National Research part of the Hungarian National Research, Development and Innovation Office (NKFIH) and TKP2021‐EGA‐24. Our research was supported by NKFI‐K‐142799 (National Research, Development and Innovation Office—A.S.), EFOP‐3.6.3‐VEKOP‐16‐2017‐00009 (F.S.), 2024‐2.1.1‐EKÖP‐2024‐00004 (D.S., F.S., D.M., and V.V), 2025‐2.1.1‐EKÖP‐2025‐00014 (D.M.), Hungarian Society of Senology (G.P.), ÚNKP‐22‐4‐I‐SE‐12 (T.D.), and the Stephen W. Kuffler Research Grant (T.D.).

## Ethics Statement

The authors have nothing to report.

## Conflicts of Interest

The authors declare no conflicts of interest.

## Supporting information




**Supporting Figure 1**: Evolution of 3D in vitro model publications based on PubMed data search (2010–2024; retrieved on February 5, 2025). A) Distribution (%) and number of scientific publications employing 3D model systems in cancer research, including organoids, spheroids, and 3D bioprinting (100% stacked column chart). B) Ratio of review articles to original research papers across different 3D model systems (stacked column chart). C) Annual percentage of review articles relative to the total number of publications (spheroids: 7.92 ± 2.47%; 3D bioprinting: 36.89 ± 7.79%; organoids: 31.45 ± 10.10% [mean ± SD]).
**Supporting Table 1**: Primary antibodies used for immunohistochemistry (IHC) and Western blot (WB) analyzes.

## Data Availability

Data generated by this study are available upon request from the corresponding author.
