# Supplementary material for: From 2D to 3D Bioprinted In Vitro Breast Cancer Model: A Comparative Study of Proliferation, Tissue Structure, and mTOR Signaling
Source: MedComm (2020). 2026 May 26;7(6):e70783. doi: 10.1002/mco2.70783 (PMC13240477; doi:10.1002/mco2.70783)
Supplement: Supplementary file 1 — Supporting Figure 1: Evolution of 3D in vitro model publications based on PubMed data search (2010–2024; retrieved on February 5, 2025). A) Distribution (%) and number of scientific publications employing 3D model systems in cancer research, including organoids, spheroids, and 3D bioprinting (100% stacked column chart). B) Ratio of review articles to original research papers across different 3D model systems (stacked column chart). C) Annual percentage of review articles relative to the total number of publications (spheroids: 7.92 ± 2.47%; 3D bioprinting: 36.89 ± 7.79%; organoids: 31.45 ± 10.10% [mean ± SD]). Supporting Table 1: Primary antibodies used for immunohistochemistry (IHC) and Western blot (WB) analyzes. [file MCO2-7-e70783-s001.docx]

Supplementary materials for

From 2D to 3D bioprinted in vitro breast cancer model: A comparative study of proliferation, tissue structure and mTOR signaling

Short title: 3D bioprinted breast cancer model

Dorottya Moldvai^1^, Gábor Petővári^1^, Rebeka Gelencsér^1^, Dániel Sztankovics^1^, Risa Miyaura^1^, Viktória Varga^1^, Fatime Szalai^1^, Kornélia Baghy^1^, Ildikó Krencz^1^, Titanilla Dankó^1^, Anna Sebestyén^1*^

^1^ Department of Pathology and Experimental Cancer Research, Semmelweis University, Üllői út 26, 1085 Budapest, Hungary

* Corresponding author: Anna Sebestyén; [sebestyen.anna@semmelweis.hu](mailto:sebestyen.anna@semmelweis.hu)

**This PDF file includes:**

Figure S1

Table S1

**Figure S1.**


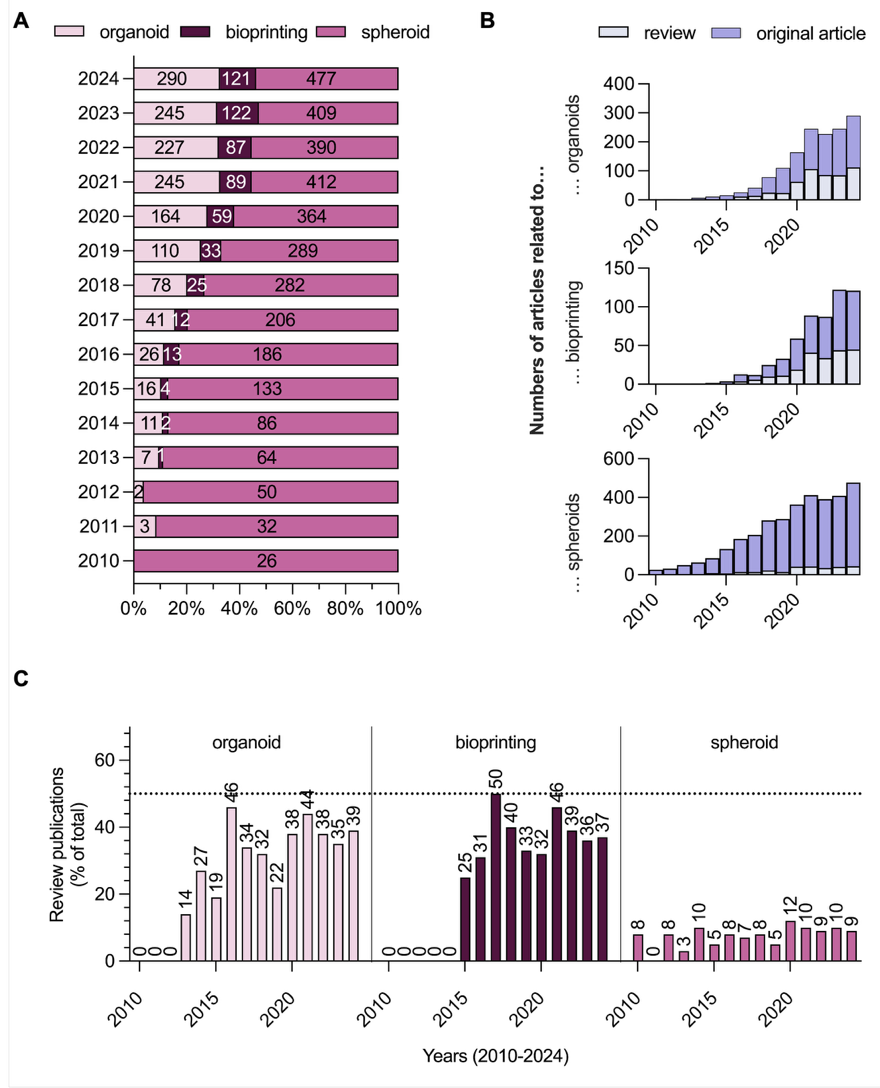


**Figure S1.** Evolution of 3D *in vitro* model publications based on PubMed data search (2010–2024; retrieved on February 5, 2025). A) Distribution (%) and number of scientific publications employing 3D model systems in cancer research, including organoids, spheroids, and 3D bioprinting (100% stacked column chart). B) Ratio of review articles to original research papers across different 3D model systems (stacked column chart). C) Annual percentage of review articles relative to the total number of publications (spheroids: 7.92 ± 2.47%; 3D bioprinting: 36.89 ± 7.79%; organoids: 31.45 ± 10.10% [mean ± SD]).

**Method**: Literature on tumor models used in cancer biology research was examined in the PubMed database using the search terms: 'cancer model' AND '3D' AND ('organoid' or 'bioprinting' or 'spheroid'). The query was made on February 5, 2025, and focused on data from 2010 to 2024, and only articles with at least an english-language abstract were included. The results were visualized in a cumulative bar chart, illustrating both the percentage distribution of the results and the growth in the number of publications over time. The ratio of review articles to original research papers was also analyzed for spheroids, organoids, and bioprinted models across the three types of 3D tumor models.

**Table S1.**

Primary antibodies used for immunohistochemistry (IHC) and Western Blot (WB) analyzes.

| **Primary antibody** | **Manufacturer** | **Cat. no.** | **Dilutions** | | **kDa** | **Target/Function/Marker** |
| --- | --- | --- | --- | --- | --- | --- |
|  |  |  | **IHC** | **WB** |  |  |
| mTOR | Cell Signaling | #2983 | 1:100 | 1:1000 | 250 | kinase of both mTORC1 and mTORC2 |
| p-(Ser2448)-mTOR | Cell Signaling | #2976 | 1:100 | - | 250 | activated form of mTOR |
|  |  | #5536 | - | 1:1000 |  |  |
| TSC1 | GeneTex | GTX130062 | - | 1:1000 | 130 | mTOR negative regulator |
| pan-Akt | Cell Signaling | #4691 | 1:500 | 1:1000 | 60 | downstream target of mTORC2 |
| p-(Ser473)-Akt | Cell Signaling | #4060 | 1:50 | 1:1000 | 60 | activated form of Akt |
| pSAPK/JNK (Thr183/Tyr185) | Cell Signaling | #4668 | - | 1:1000 | 54/46 | stress response/kinase |
| S6 | Cell Signaling | #2317 | 1:100 | 1:1000 | 32 | downstream target of mTORC1 |
| p-(Ser235/236)-S6 | Cell Signaling | #4858 | 1:100 | 1:1000 | 32 | activated form of S6 |
| p-(Ser240/244)-S6 | Cell Signaling | #2215 | - | 1:1000 | 32 | activated form (phosphorylated only through mTOR) |
| Rictor | Bethyl Laboratories | #A500-002A | 1:1000 | - | 200 | mTORC2 scaffold protein |
|  | Cell Signaling | #2140 | - | 1:1000 |  |  |
| Raptor | Abcam | #ab40768 | 1:100 | 1:1000 | 150 | mTORC1 scaffold protein |
| cleaved-caspase-3 | Cell Signaling | #9664 | 1:100 | - | - | apoptosis |
| LC3 | Cell Signaling | #3868 | 1:100 | - | - | autophagy |
| Ki67 | ThermoFischer | #PA5-19462 | 1:200 | - | - | proliferation |
| N-cadherin | BD Biosciences | #610921 | 1:100 | - | - | cell adhesion |
| E-cadherin | GeneTex | #GTX629691 | 1:100 | - | - | cell adhesion |
| β-catenin | Merck | #224M-14 | 1:50 | - | - | cell-cell adhesion and transcription factor |
| fibronectin | ThermoFischer | #MS-1351 | 1:100 | - | - | epithelial-mesenchymal transition |
| syndecan 1 | SinoBiological | 50641-RP02 | 1:500 | - | - | cell adhesion |
| β-actin | Sigma-Aldrich | #A2228 | - | 1:5000 | 45 | cytoskeleton/loading control |
